# Kinetics and thermodynamics dataset of iron oxide reduction using torrefied microalgae for chemical looping combustion

**DOI:** 10.1016/j.dib.2020.105261

**Published:** 2020-02-07

**Authors:** Aristotle T. Ubando, Wei-Hsin Chen, Veeramuthu Ashokkumar, Jo-Shu Chang

**Affiliations:** a Department of Aeronautics and Astronautics, National Cheng Kung University, Tainan, 701, Taiwan; b Mechanical Engineering Department, De La Salle University, 2401 Taft Avenue, 0922, Manila, Philippines; c Department of Chemical and Materials Engineering, College of Engineering, Tunghai University, Taichung, 407, Taiwan; d Department of Mechanical Engineering, National Chin-Yi University of Technology, Taichung, 411, Taiwan; e Research Center for Energy Technology and Strategy, National Cheng Kung University, Tainan, 701, Taiwan; f Department of Chemical Engineering, National Cheng Kung University, Tainan, 701, Taiwan

**Keywords:** Iron oxide reduction, Microalage, Kinetics, Thermodynamics, Torrefaction, Chemical looping combustion, Ironmaking

## Abstract

The reduction of iron oxides transpires through the application of heat wherein a carbon source known as reductant is required. In order to design a chemical looping combustion using iron as an oxygen carrier and torrefied microalgae biomass as a reductant, the kinetics and thermodynamics dataset must be determined. Using the Arrhenius law of reaction, the kinetics dataset was obtained employing the three chemical reaction model such as the first order (C1), the reaction order 1.5 (C1.5), and the second-order (C2). The iron oxide reduction from hematite to metallic iron was sub-divided into three phases wherein phase 1 (Fe_2_O_3_ → Fe_3_O_4_) is from 365 °C to 555 °C, phase 2 (Fe_3_O_4_ → FeO) is from 595 °C to 799 °C, and phase 3 (FeO → Fe) is from 800 °C to 1200 °C. Two torrefied microalgae (Chlamydomonas sp. JSC4 and Chlorella vulgaris ESP-31) were considered as a reducing agent. The kinetics dataset comprise of the activation energy (E), pre-exponential factor (A), and the reaction rate (k) while the thermodynamic dataset consists of the change in enthalpy (ΔH), change in Gibbs energy (ΔG), and change in entropy (ΔS). These kinetics and thermodynamics parameters are essential in understanding the reaction mechanisms of the reduction process of iron oxides enabling process optimization and improvement. Current literature lacks the kinetics and thermodynamics datasets for the reduction of iron oxides using the two torrefied microalgae as reductants. This work provides these datasets which are useful for the design of reactors in chemical looping combustion.

**Table:** Specifications Table

Subject	Energy (General)
Specific subject area	Iron oxide reduction, Kinetics, Thermodynamics
Type of data	Tables and figures
How data were acquired	The data presented is obtained from the experimental data of the reduction process between hematite and two torrefied microalgal biomass from Ubando et al. [1]. Using the Arrhenius law, the kinetic parameters such as the activation energy (E_a_), pre-exponential factor (A), and the reaction rate (k) were obtained. In addition, the thermodynamic parameters of the reaction were quantified.
Data format	Raw and analyzed
Parameters for data collection	The considered parameters for the dataset include the use of two torrefied microalgal biomass which are the Chlamydomonas sp. JSC4 and Chlorella vulgaris ESP-31 in the iron oxide reduction. In addition, the varying ratio of the hematite and the carbon source was also varied between 1:1 and 2:1.
Description of data collection	The raw data from the thermogravimetric analysis from Ubando et al. [1] was used as input data for the kinetics and thermodynamics analysis. The kinetic parameters obtained are composed of the activation energy and the pre-exponential factor of the reaction. Moreover, the thermodynamic parameters include the change in enthalpy in the reaction, the Gibbs free energy, and the change in entropy in the reaction. The model fit was determined using the R^2^ in the linear regression.
Data source location	The data was generated in the GenFuel Laboratory at the Department of Aeronautics and Astronautics, National Cheng Kung University, Tainan, Taiwan. The data is in this article.
Data accessibility	All the data can be accessed through this article.
Related research article	Author's name: Aristotle T. Ubando, Wei-Hsin Chen, Veeramuthu Ashokkumar, Jo-Shu Chang Title: Iron oxide reduction by torrefied microalgae for CO2 capture and abatement in chemical-looping combustion Journal: Energy https://doi.org/10.1016/j.energy.2019.115903

**Table:** 

**Value of the Data** • The thermogravimetric data from Ubando et al. [1] to describe the iron oxide reduction was utilized as input data for the kinetic and thermodynamic analysis. • The three phases of iron oxide reduction using torrefied microalgae biomass were identified. • The kinetics parameters were quantified using Coats-Redfern model employing three chemical reaction model orders for the three phases of iron oxide reduction. • The thermodynamic parameters of the three phases of iron oxide reduction were determined. • The data is useful for researchers and engineers looking for the reaction mechanism of the iron oxide reduction using torrefied microalgae biomass. • The kinetic and thermodynamic parameters of the iron oxide reduction are useful for designing reactors for biomass-based chemical looping combustion.

## 1 Data description

The dataset in this article describes the iron oxide reduction of hematite using torrefied microalgae biomass as a reductant for the three reduction phase. The figures and tables are described as follows.

Table 1 shows the various types of chemical reaction order and the corresponding f(α) and g(α). Table 2 illustrates the R^2^ for the linear regression model fit for the three phases on the various blends of the hematite and the torrefied microalgae biomass. Table 3 exhibits the kinetics dataset of the iron oxide reduction for the three phases, three chemical reaction model order, and the different blends of the hematite and the torrefied microalgae biomass. Table 4 shows the thermodynamic dataset of the iron oxide reduction for the three phases, three chemical reaction model order, and the different blends of the hematite and the torrefied microalgae biomass.

**Table 1:** The various types of chemical reaction order and the corresponding f(α) and g(α).

Model description	f(α)	g(α)
First order (C1)	(1- α)^n^, n = 1	−ln(1−α)
Reaction order 1.5 (C1.5)	(1- α)^n^, n = 3/2	2[(1−α) ^−3/2^−1]
Second order (C2)	(1- α)^n^, n = 2	(n−1)^−1^(1−α)^(1−n)^

**Table 2:** The R^2^ for the linear regression model fit for the three phases.

Material	Ratio type	Model type	Phase 1 (365–555°C)	Phase 2 (595–799°C)	Phase 3 (800–1200 °C)
Hematite-Chlamydomonas	1:1 ratio	C1	0.9109	0.8927	0.9524
		C1.5	0.9466	0.8961	0.8853
		C2	0.9110	0.9160	0.9043
	2:1 ratio	C1	0.9079	0.8917	0.9534
		C1.5	0.9445	0.8982	0.8744
		C2	0.9168	0.9142	0.8995
Hematite-Chlorella vulgaris	1:1 ratio	C1	0.9099	0.8925	0.9593
		C1.5	0.9460	0.8917	0.8699
		C2	0.9117	0.9139	0.9019
	2:1 ratio	C1	0.9087	0.8982	0.9701
		C1.5	0.9433	0.9086	0.8482
		C2	0.9109	0.9175	0.8957

**Table 3:** The kinetics dataset of the iron oxide reduction.

Material	Ratio type	Model type	Phase 1 (365–555 °C)			Phase 2 (595–799°C)			Phase 3 (800–1200 °C)		
			E_a_ (kJ mol^−1^)	A (min^−1^)	k (min^−1^)	E_a_ (kJ mol^−1^)	A (min^−1^)	k (min^−1^)	E_a_ (kJ mol^−1^)	A (min^−1^)	k (min^−1^)
Hematite-Chlamydomonas	1:1 ratio	C1	98.21	1.55 × 10^18^	1.60 × 10^11^	161.92	1.51 × 10^20^	2.89 × 10^11^	181.92	1.34 × 10^19^	1.19 × 10^11^
		C1.5	1159.88	1.35 × 10^233^	3.96 × 10^150^	1294.82	7.66 × 10^258^	1.49 × 10^189^	1399.39	5.83 × 10^278^	6.53 × 10^216^
		C2	22.43	3.18 × 10^14^	8.06 × 10^12^	22.51	5.22 × 10^14^	3.20 × 10^13^	23.27	1.23 × 10^15^	1.15 × 10^14^
	2:1 ratio	C1	97.40	1.33 × 10^18^	1.56 × 10^11^	162.68	1.70 × 10^20^	2.97 × 10^11^	194.68	4.39 × 10^19^	1.06 × 10^11^
		C1.5	1167.62	4.07 × 10^234^	3.37 × 10^151^	1294.57	6.86 × 10^258^	1.39 × 10^189^	1457.33	6.24 × 10^289^	1.97 × 10^225^
		C2	22.29	3.04 × 10^14^	7.89 × 10^12^	22.53	5.29 × 10^14^	3.24 × 10^13^	23.47	1.29 × 10^15^	1.18 × 10^14^
Hematite-Chlorella vulgaris	1:1 ratio	C1	99.42	1.92 × 10^18^	1.61 × 10^11^	160.31	1.18 × 10^20^	2.76 × 10^11^	188.07	2.14 × 10^19^	1.02 × 10^11^
		C1.5	1163.43	6.34 × 10^233^	1.01 × 10^151^	1308.00	2.54 × 10^261^	9.67 × 10^190^	1449.23	1.86 × 10^288^	1.35 × 10^224^
		C2	22.49	3.25 × 10^14^	8.16 × 10^12^	22.52	5.20 × 10^14^	3.19 × 10^13^	23.37	1.23 × 10^15^	1.14 × 10^14^
	2:1 ratio	C1	99.73	1.97 × 10^18^	1.58 × 10^11^	167.85	3.54 × 10^20^	3.25 × 10^11^	193.70	2.96 × 10^19^	7.90 × 10^10^
		C1.5	1170.68	1.53 × 10^235^	7.84 × 10^151^	1272.94	4.29 × 10^254^	1.45 × 10^186^	1522.18	1.54 × 10^302^	6.44 × 10^234^
		C2	22.50	3.27 × 10^14^	8.19 × 10^12^	22.49	5.24 × 10^14^	3.22 × 10^13^	23.72	1.29 × 10^15^	1.15 × 10^14^

**Table 4:** The thermodynamic dataset of iron oxide reduction.

Material	Ratio type	Model type	Phase 1 (365–555°C)			Phase 2 (595–799°C)			Phase 3 (800–1200°C)		
			ΔH (kJ mol^−1^)	ΔG (kJ mol^−1^)	ΔS (kJ mol^−1^ K^−1^)	ΔH (kJ mol^−1^)	ΔG (kJ mol^−1^)	ΔS (kJ mol^−1^ K^−1^)	ΔH (kJ mol^−1^)	ΔG (kJ mol^−1^)	ΔS (kJ mol^−1^ K^−1^)
Hematite-Chlamydomonas	1:1 ratio	C1	92.10	−31.45	0.091	153.85	−16.05	0.126	172.11	145.07	0.081
		C1.5	1153.78	−4536.00	4.206	1286.75	−5064.87	4.695	1389.58	−305.19	5.051
		C2	16.32	−11.69	0.021	14.45	−14.04	0.021	13.46	12.34	0.003
	2:1 ratio	C1	91.30	−30.53	0.090	154.61	−16.64	0.127	184.87	153.56	0.091
		C1.5	1161.52	−4566.57	4.234	1286.51	−5063.87	4.694	1447.51	−360.85	5.263
		C2	16.19	−11.33	0.020	14.46	−14.19	0.021	13.66	12.16	0.004
Hematite-Chlorella vulgaris	1:1 ratio	C1	93.32	−32.61	0.093	152.24	−14.91	0.124	178.26	149.72	0.085
		C1.5	1157.33	−4549.86	4.219	1299.93	−5116.97	4.744	1439.41	−325.60	5.233
		C2	16.39	−11.89	0.021	14.46	−14.00	0.021	13.56	12.40	0.003
	2:1 ratio	C1	93.63	−32.60	0.093	159.79	−19.72	0.133	183.89	153.25	0.088
		C1.5	1164.58	−4578.41	4.245	1264.87	−4978.18	4.615	1512.37	−398.94	5.500
		C2	16.39	−11.93	0.021	14.42	−14.11	0.021	13.91	12.29	0.005

**Fig. 1:**
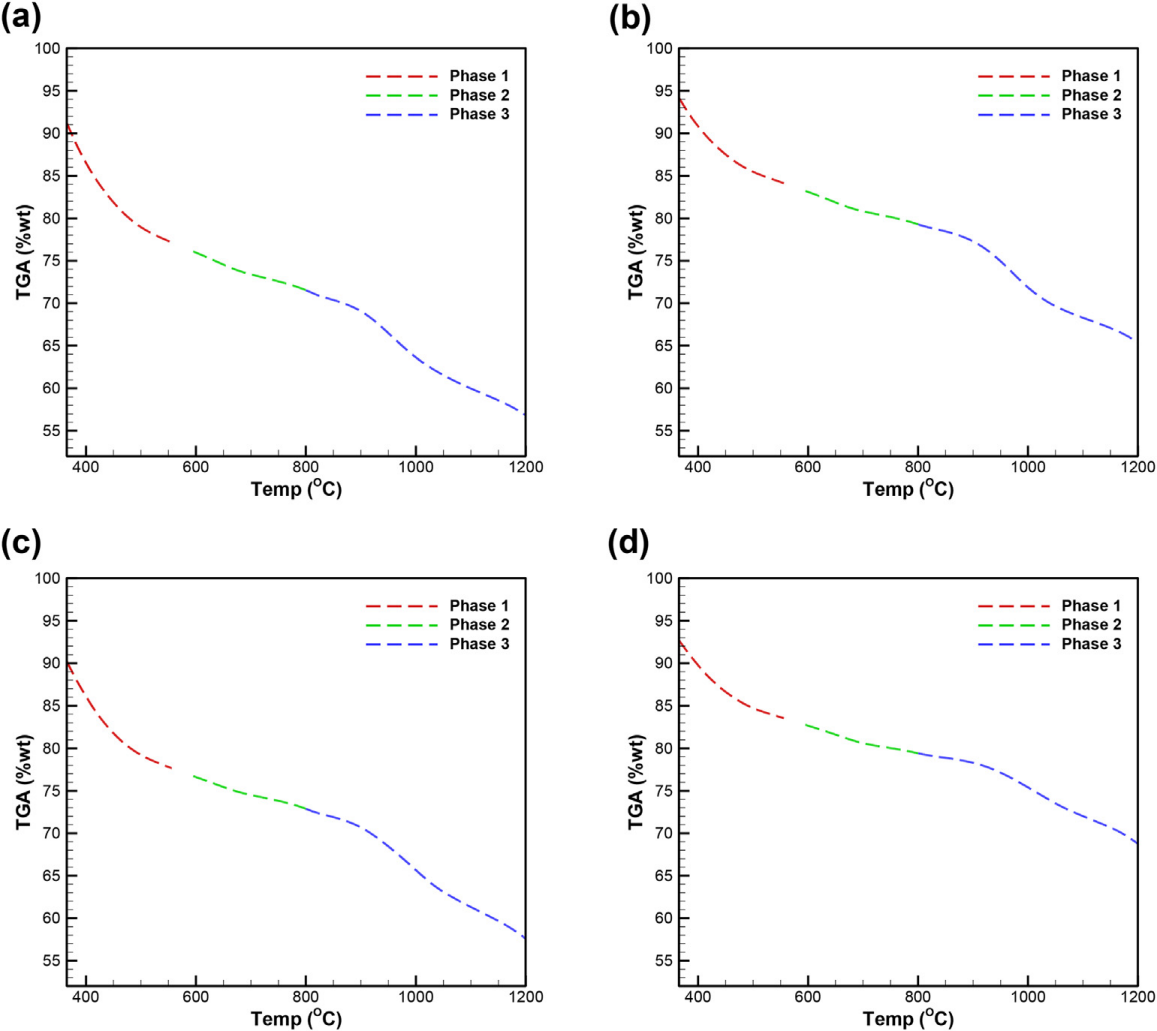
Thermogravimetric data for and its iron oxide reduction phases for the blends of a) hematite and Chlamydomonas sp. JSC4 at 1:1 ratio, b) hematite and Chlamydomonas sp. JSC4 at 2:1 ratio, c) hematite and Chlorella vulgaris ESP-31 at 1:1 ratio, d) hematite and Chlorella vulgaris ESP-31 at 2:1 ratio (adopted from Ubando et al. [1]).

**Fig. 2:**
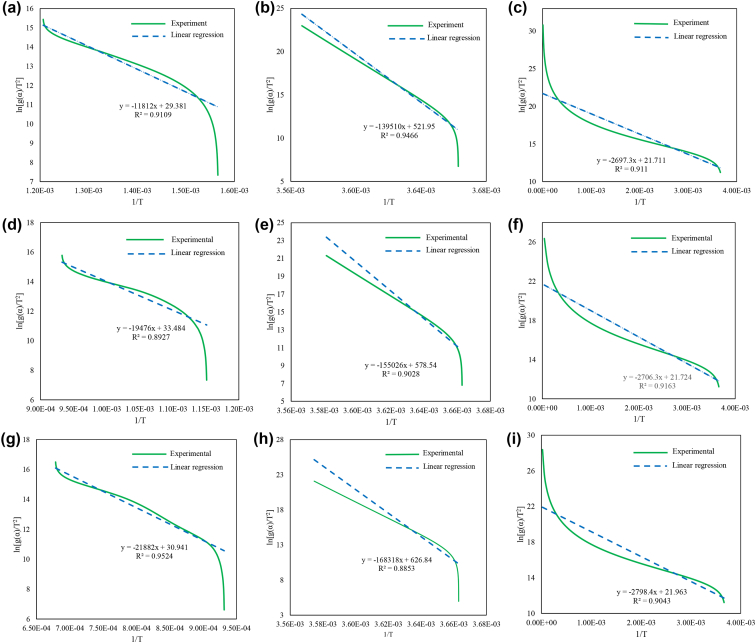
The model fits on the 1:1 blend of hematite and Chlamydomonas sp. JSC4 for a) Phase 1 and C1, b) Phase 1 and C1.5, c) Phase 1 and C2, d) Phase 2 and C1, e) Phase 2 and C1.5, f) Phase 2 and C2, g) Phase 3 and C1, h) Phase 3 and C1.5, and i) Phase 3 and C2.

**Fig. 3:**
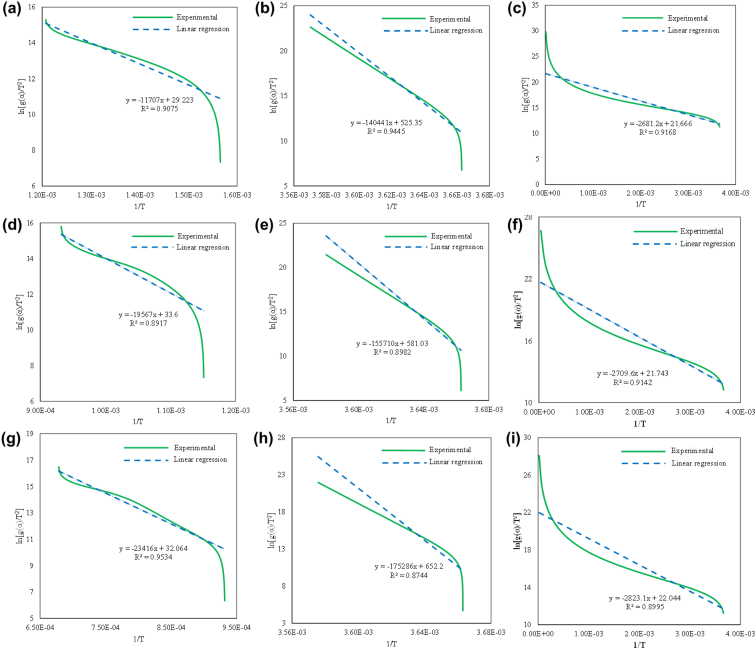
The model fits on the 2:1 blend of hematite and Chlamydomonas sp. JSC4 for a) Phase 1 and C1, b) Phase 1 and C1.5, c) Phase 1 and C2, d) Phase 2 and C1, e) Phase 2 and C1.5, f) Phase 2 and C2, g) Phase 3 and C1, h) Phase 3 and C1.5, and i) Phase 3 and C2.

**Fig. 4:**
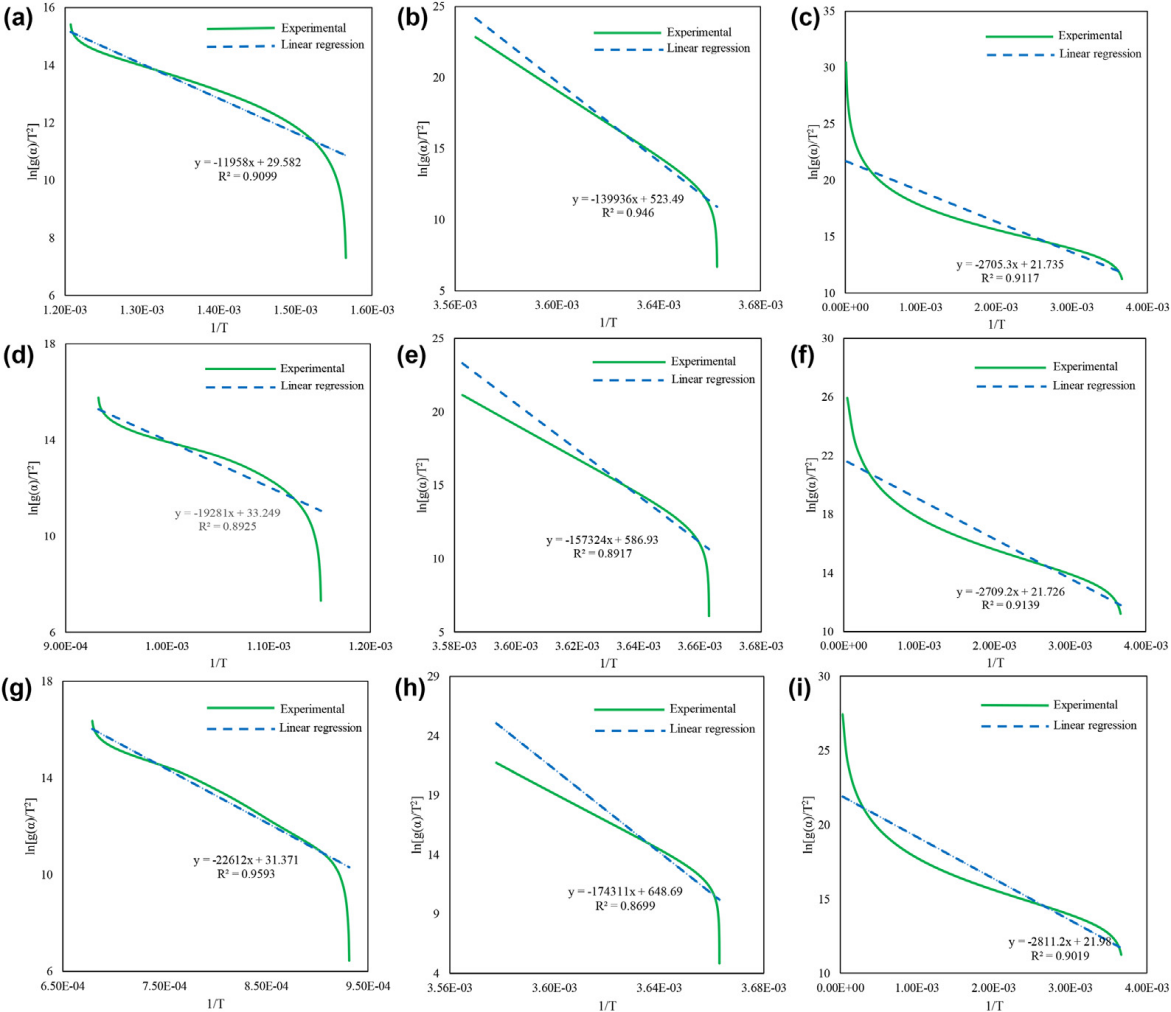
The model fits on the 1:1 blend of hematite and Chlorella vulgaris ESP-31 for a) Phase 1 and C1, b) Phase 1 and C1.5, c) Phase 1 and C2, d) Phase 2 and C1, e) Phase 2 and C1.5, f) Phase 2 and C2, g) Phase 3 and C1, h) Phase 3 and C1.5, and i) Phase 3 and C2.

**Fig. 5:**
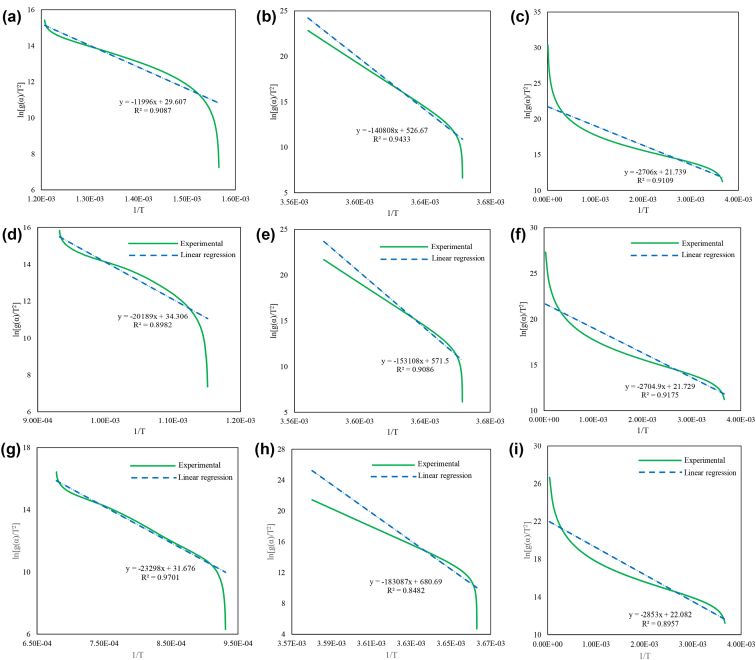
The model fits on the 2:1 blend of hematite and Chlorella vulgaris ESP-31 for a) Phase 1 and C1, b) Phase 1 and C1.5, c) Phase 1 and C2, d) Phase 2 and C1, e) Phase 2 and C1.5, f) Phase 2 and C2, g) Phase 3 and C1, h) Phase 3 and C1.5, and i) Phase 3 and C2.

Fig. 1 identifies the three iron reduction phases through the thermogravimetric dataset from Ubando et al. [1] for the two microalgae species. Fig. 2 describes the model fit on the 1:1 blend of hematite and Chlamydomonas sp. JSC4 for the three phases and three chemical reaction model order. Fig. 3 describes the model fit on the 2:1 blend of hematite and Chlamydomonas sp. JSC4 for the three phases and three chemical reaction model order. Fig. 4 describes the model fit on the 1:1 blend of hematite and Chlorella vulgaris ESP-31 for the three phases and three chemical reaction model order. Fig. 5 describes the model fit on the 2:1 blend of hematite and Chlorella vulgaris ESP-31 for the three phases and three chemical reaction model order.

## 2 Experimental design, materials, and methods

### 2.1 The iron oxide reduction

The iron oxide reduction of hematite with torrefied microalgae biomass as a carbon source was conducted by Ubando et al. [1] using a PerkinElmer Diamond thermogravimetric and differential analyzer (TGA). The TGA settings are described by Ubando et al. [1] in detail. The two microalgae species considered as a carbon source were Chlamydomonas sp. JSC4 [2] and Chlorella vulgaris ESP-31 [3]. The two microalgae biomass species were prepared using the torrefaction process described in Ubando et al. [1] to produce the torrified microalgae biomass. The three phases of iron oxide reduction were identified as phase 1 from 365 °C to 555 C for hematite (Fe_2_O_3_) to magnetite (Fe_3_O_4_), phase 2 from 595 C to 799°C for magnetite (Fe_3_O_4_) to wustite (FeO), and phase 3 from 800 C to 1200°C for wustite (FeO) to metallic iron (Fe) [1]. The TGA data of the three iron oxide reduction phases from Ubando et al. [1] is shown in Fig. 1. The TGA data shown in Fig. 1 is used as input data to the kinetic and thermodynamic analysis of the three iron oxide reduction phases.

### 2.2 The kinetics dataset

The kinetics dataset for the three phases was determined using the Arrhenius law of reaction which employed the Coats-Redfern model for the integral approximation and thermodynamic analysis described by Naqvi et al. [4]. The kinetics dataset was quantified using the LINEST formula in MS Excel. The three different chemical reaction model orders are shown in Table 1.

The kinetics dataset was identified using the Arrhenius law which as shown in Eqs. (1)–(4).

(1) $$\frac{d\alpha }{dt}=k\left(T\right)f\left(\alpha \right)$$

(2) $$\alpha =\frac{m_{o}-m_{i}}{m_{o}-m_{f}}$$

(3) $$k\left(T\right)=A\;exp\left(-\frac{E_{a}}{RT}\right)$$

(4) $$\frac{d\alpha }{dT}=\frac{A}{\beta }exp\left(-\frac{E_{a}}{RT}\right)$$

where the conversion degree is defined by the factor α, the conversion time t, k is the reaction rate, the initial and the final mass of the sample are described by m_i_, m_o_, and m_f_, respectively. In addition, A is the pre-exponential factor (min^−1^), Ea is the activation energy (kJ mol-1), R is the universal gas constant (=0.008314 kJ mol^−1^ K^−1^), and T is the reaction temperature (K).

In order to solve for the unknowns with the integral of Eq. (4), the integral form of the reaction model g(α) is introduced using the Coats-Redfern model to approximate the kinetic parameters described in Naqvi et al. [4] as shown in Eq. (5).

(5) $$ln\left[\frac{g\left(\alpha \right)}{T^{2}}\right]=ln\frac{AR}{\beta E_{a}}\left(1-\frac{2RT}{E_{a}}\right)-\frac{E_{a}}{RT}$$

where g(α) is the kinetic function of different reaction models.

The conversion degree α is determined through the TGA data provided in Fig. 1 and using Eq. (2) for each iron oxide reduction phase. Once the conversion degree has been quantified, the expression ln [g(α)/T^2^] is determined through the relation of the chemical reaction model shown in Table 1 and the quantification of 1/T. For each iron oxide reduction phase, the slope is determined by fitting the line between ln [g(α)/T^2^] and 1/T using the LINEST formula. The summary of the R^2^ model fit for the three reaction models on the three phases of reduction is shown in Table 2. The summary of the kinetics dataset of the iron oxide reduction for the three phases such as the activation energy (E_a_), pre-exponential factor (A), and rate of reaction (k) are shown in Table 3.

The model fit for the iron oxide reduction of the 1:1 blend of hematite and Chlamydomonas sp. is shown in Fig. 2, the 2:1 blend of hematite and Chlamydomonas sp. is shown in Fig. 3, the 1:1 blend of hematite and Chlorella vulgaris is shown in Fig. 4, and the 2:1 blend of hematite and Chlorella vulgaris is shown in Fig. 5.

From the linear equations shown in Fig. 2, Fig. 3, Fig. 4, Fig. 5 for the various blends of hematite and torrefied microalgae biomass and the three iron reduction phases, the kinetic dataset is solved using the linear relationship shown in Eq. (5).

### 2.3 The thermodynamics dataset

The thermodynamics dataset was established using the following relation as described in Naqvi et al. [4].

(6) $$\text{Δ}H=E_{a}-RT$$

(7) $$\text{Δ}G=E_{a}+RT_{m}ln\left(\frac{K_{B}T}{hA}\right)$$

(8) $$\text{Δ}S=\frac{\text{Δ}H-\text{Δ}G}{T_{m}}$$

where *h* is the Planks constant (=6.626 × 10^−34^ m^2^ kg s^−1^), *K*_*B*_ is the Boltzmann constant (=1.381 × 10^−23^m^2^ kg s^−2^ K^−1^), and *T*_*m*_ is the maximum temperature with maximum thermogravimetric degradation. Using Eqs. (6), (7), (8), the thermodynamic dataset was determined and is shown in Table 4.
